# Total hip arthroplasty: leg length inequality impairs functional outcomes and patient satisfaction

**DOI:** 10.1186/1471-2474-13-95

**Published:** 2012-06-11

**Authors:** Christoph Röder, Raphael Vogel, Lukas Burri, Daniel Dietrich, Lukas P Staub

**Affiliations:** 1 Institute for Evaluative Research in Orthopaedics, University of Bern, Stauffacherstrasse 78, 3014, Bern, Switzerland; 2 Institute for Mathematical Statistics and Actuarial Science, University of Bern, Sidlerstrasse 5, 3012, Bern, Switzerland

## Abstract

**Background:**

Leg length inequality (LLI) was identified as a problem of total hip arthroplasty soon after its introduction. Leg lengthening is the most common form of LLI. Possible consequences are limping, neuronal dysfunction and aseptic component loosening. LLI can result in an increased strain both on the contralateral hip joint and on the abductor muscles. We assessed the influence of leg lengthening and shortening on walking capacity, hip pain, limping and patient satisfaction at 2-year follow-up.

**Methods:**

478 cases with postoperative lengthening and 275 with shortening were identified, and matched with three controls each. Rigorous adjustment for potential differences in baseline patient characteristics was performed by propensity-score matching of covariates. The arbitrarily defined desired outcomes were a walking capacity >60 minutes, no hip pain, no limping, and excellent patient satisfaction. Differences in not achieving the desired outcomes between the groups were expressed as odds ratios.

**Results:**

In the lengthened case group, the odds ratio for not being able to walk for an hour was 1.70 (95% CI 1.28-2.26) for cases compared to controls, and the odds ratio for having hip pain at follow-up was 1.13 (95% CI 0.78-1.64). The odds ratio for limping was 2.08 (95% CI 1.55-2.80). The odds ratio for not achieving excellent patient satisfaction was 1.67 (95% CI 1.23-2.28). In the shortening case group, the odds ratio for not being able to walk for an hour was 1.23 (95% CI 0.84-1.81), and the odds ratio for having hip pain at follow-up was 1.60 (95% CI 1.05-2.44). The odds ratio for limping for cases was 2.61 (95% CI 1.78-3.21). The odds ratio for not achieving excellent patient satisfaction was 2.15 (95% CI 1.44-3.21).

**Conclusions:**

Walking capacity, limping and patient satisfaction were all significantly associated with leg lengthening, whereas pain alleviation was not. In contrast, hip pain, limping and patient satisfaction were all significantly associated with leg shortening, whereas walking capacity was not.

## Background

Total hip arthroplasty (THA) is one of the most commonly performed orthopaedic interventions today. Due to new operating techniques, new materials and operative planning methods, surgical outcomes have constantly been improving since the beginning of hip replacement surgery. The favourable long term results of THA support its expanded role in modern orthopaedic surgery, particularly in view of the steadily growing prevalence of degenerative hip disease around the world [1,2].

Although relief of pain and improvement of function are the main objectives of THA, the maintenance or re-establishment of equal leg length is highly desirable. Postoperative leg length inequality is not only a bothersome complication but also one of the main reasons for lawsuits after THA in the United States [3]. An excellent clinical result with respect to pain relief and radiographic appearance must be considered a surgical failure if patients are dissatisfied because of leg length inequality leading to functional deficiencies. To avoid disturbance of the static and dynamic equilibrium of the loco-motor system, these patients may require a shoe lift to lessen postoperative limping. Even so, research on the impact of a postoperative leg length discrepancy on patient function and satisfaction is scarce.

With two cohort studies (leg lengthening, leg shortening) we therefore set out to assess whether patients exposed to leg length inequality after THA showed impaired physical function and satisfaction compared to patients with equally long legs.

## Methods

### Measurement of outcomes and characteristics of the cohort

The two cohort studies were nested in the prospective data collection of the IDES (International Documentation and Evaluation System) hip registry of the Institute for Evaluative Research in Orthopaedics, formerly Maurice E. Müller Center for Education and Documentation. The history and administration of the IDES registry have been described in detail [4]. In short, dedicated content report forms, completed by orthopaedic surgeons, were used to collect perioperative information about patient history, clinical examination and surgical intervention. In the same manner, clinical outcomes such as walking capacity (>60 min, 31-60 min, 13-30 min, <10 min), hip pain (none, mild, moderate, severe, intolerable), limp without support (none, slight, moderate, severe), and patient satisfaction (excellent, good, fair, poor) were recorded by surgeons at follow-up examinations. For the purpose of this study, we arbitrarily defined the following desired outcomes: walking capacity >60 minutes, no hip pain, no limping, and excellent patient satisfaction. The current study did not require institutional review board approval at our center, as it utilised existing anonymous observational data.

**Figure 1:**
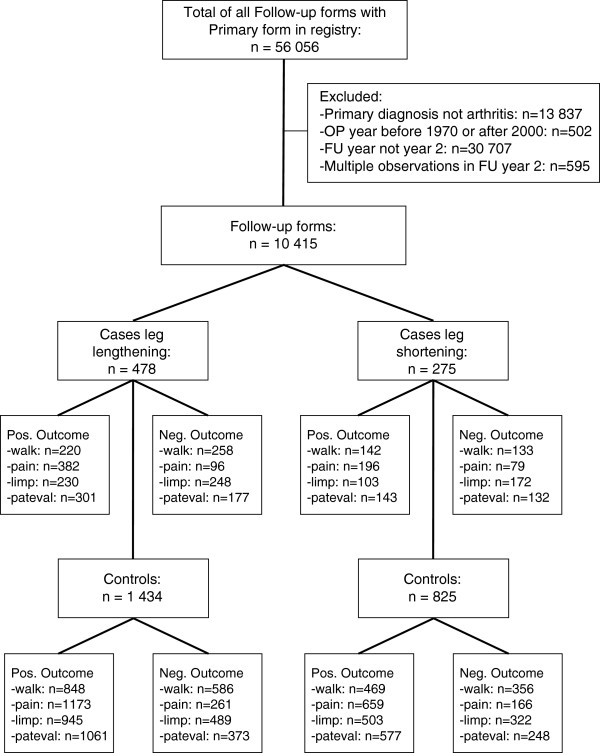
Study profile of included patients in the two cohorts.

Patients operated on from 1970 through 2000 with a diagnosis of osteoarthritis of the hip and preoperative equal leg lengths were included. No exclusions based upon status of contralateral hip, locomotor system or comorbid conditions were made. To minimise the influence of soft tissue contractures, which can lead to functional leg length inequality lasting up to 6 months postoperative, only patients with a documented follow-up examination in follow-up year two were included for outcome assessment [5]. In cases of multiple follow-up examinations in year two, the one closest to the middle of the two-year interval after surgery was chosen. The selection process of the study samples is shown in Figure 1. The query resulted in 10`415 datasets with complete baseline and follow-up information. The data were derived from 15 centers in four European countries (Switzerland, Germany, France, Italy). 

Leg length inequality was reported on IDES forms in steps of 1 cm. The method used to measure leg length inequality was at the discretion of the orthopaedic surgeon but was not recorded on the forms. Patients with a reported leg lengthening after THA were identified as cases for the first study, and those with leg shortening were identified as cases for the second study. Leg lengthening was defined as a lengthening of the operated leg of at least one centimetre. Similarly, leg shortening was defined as a shortening of the operated leg of at least one centimetre. Furthermore, cases were divided into two subgroups with a leg lengthening/shortening of one centimetre or more than one centimetre, respectively. We chose these values because it has been shown that significant symptoms become apparent with leg length inequality of at least one centimetre [6,7].

The control groups consisted of patients with no documented leg lengthening after THA. In order to reduce selection bias and potential confounding in these observational studies, we adjusted for potential differences in the baseline characteristics of patients with the use of propensity-score matching of covariates. Three controls were matched to each case. Factors expected to have an influence on the outcome include sex, age at operation, hip pain and time walked without support before the operation, decade of intervention and materials used. For detailed information on matching characteristics see Tables 1 and 2.

**Table 1:** Leg lengthening: baseline patient characteristics after matching

**Baseline characteristics**		**Controls (N = 1434)**	**Cases (N = 478)**
**Sex**	M	46.7%	46.7%
	F	53.3%	53.3%
**Age at procedure (years)**	Mean (SD)	67 (9)	67 (9)
**Weight (kg)**	Mean (SD)	74 (14)	75 (15)
**Height (cm)**	Mean (SD)	165 (9)	165 (9)
**Charnley class**	A	54.3%	47.4%
	BB	22.4%	29.6%
	B	21.4%	21.1%
	C	1.9%	1.9%
**Previous Surgery**	Yes	3.6%	4.4%
**Clinic Size**	small	5.0%	5.0%
	medium	52.4%	49.2%
	large	42.6%	45.8%
**Fixation of Components**	all cemented	17.5%	16.3%
	all uncemented	18.0%	18.8%
	hybrid	50.0%	49.2%
	reverse hybrid	14.5%	15.7%
**Duration of procedure**	<90 min	54.5%	55.4%
	90 min > x < 135 min	38.6%	37.7%
	>135 min	6.9%	6.9%
**Decade of procedure**	1970's	3.0%	1.5%
	1980's	50.8%	50.0%
	1990's	45.3%	47.7%
	after 2000	0.9%	0.8%

Abbreviation: SD = standard deviation.

**Table 2:** Leg shortening: baseline patient characteristics after matching

**Baseline characteristics**		**Controls (N = 825)**	**Cases (N = 275)**
**Sex**	M	50.2%	50.2%
	F	49.8%	49.8%
**Age at procedure (years)**	Mean (SD)	67 (9)	67 (9)
**Weight (kg)**	Mean (SD)	75 (14)	75 (13)
**Height (cm)**	Mean (SD)	167 (9)	167 (9)
**Charnley class**	A	59.7%	62.2%
	BB	17.5%	16.8%
	B	20.9%	18.9%
	C	1.9%	2.1%
**Previous Surgery**	Yes	4.0%	4.7%
**Clinic Size**	small	10.1%	10.5%
	medium	32.6%	26.6%
	large	57.3%	62.9%
**Fixation of components**	all cemented	18.3%	16.3%
	all uncemented	17.8%	16.7%
	hybrid	50.2%	50.6%
	reverse hybrid	13.7%	16.4%
**Duration of procedure**	<90 min	46.9%	48.0%
	90 min > x <135 min	40.9%	42.9%
	>135 min	12.2%	9.1%
**Decade of procedure**	1970's	5.2%	1.1%
	1980's	44.5%	48.0%
	1990's	49.0%	49.8%
	after year 2000	1.3%	1.1%

Abbreviation: SD = standard deviation.

### Statistical analysis

The primary outcome of this study was to compare patients with and without leg length inequality in terms of their walking capacity, hip pain, limping and satisfaction with outcome. A number of covariates for each patient (e.g. sex, age at operation, clinic size, decade of surgery) were used to adjust for confounding using the propensity score method, as described in detail by Rosenbaum and Rubin [8]. In brief, an individual’s propensity score is defined as his or her conditional probability of being exposed to leg length inequality versus equally long legs, given the observed covariates. Hence, two patients with the same propensity score have an equal estimated probability of exposure. If one was exposed and the other unexposed, the exposure allocation could be considered random, conditional on the observed covariates. Therefore, akin to a randomised controlled trial (RCT), there is balance of the covariates between exposure groups after adjusting for the propensity score. There is, however, an important difference between propensity score adjustment and RCTs, in that the latter is able to balance both measured and unmeasured covariates. Propensity scores can only control for the measured covariates. Propensity scores have been used in psychiatric and cardiological research [9-11], but they have not yet been applied in orthopaedic research.

The individual propensity scores were obtained from a multiple logistic regression model and were then fed into a greedy matching algorithm in order to match three controls (equal leg length) to each case (patients with leg length inequality). Differences in achieving the desired outcomes between the groups were then expressed as odds ratios using generalised estimation equation (GEE) models. To ensure an overall significance level of 0.05, Bonferroni correction was used to adjust for multiple testing in the four endpoints, and adjusted p-values (p_a_-values) and adjusted 95% confidence intervals (95% aCI) are presented throughout the paper. All statistical analyses were conducted using SAS 9.2 (SAS Institute Inc, Cary, NC).

## Results

### Leg lengthening

#### Patient characteristics leg lengthening

From a total of 10'415 potential follow-up examinations, 478 cases with leg lengthening were identified, comprising 405 patients with a lengthening of 1 cm and 73 patients with a lengthening of more than 1 cm. To these cases, 1`434 controls were matched. As shown in Table 1, propensity-score matching achieved a nearly equal distribution of overt pre-operative patient characteristics between cases and controls.

#### Influence of leg lengthening on patient outcomes

At follow-up two years after the intervention, 54% (258 patients) in the case group were not able to walk for more than 60 minutes without support, compared to 41% (586 patients) in the control group. Hip pain was reported in 20% (96 patients) of cases and 18% (261 patients) of controls. Limping was seen in 52% (248 patients) of cases and 34% (489 patients) of controls. Also, 37% (177 patients) in the case group did not rate their subjective patient satisfaction as excellent, in contrast to 26% (373 patients) of the controls.

**Figure 2:**
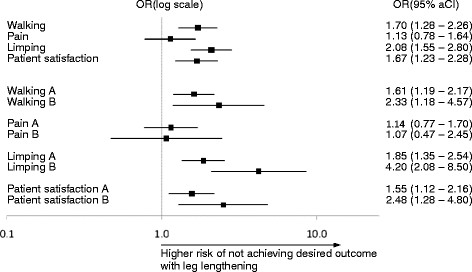
Odds ratios and 95% adjusted confidence intervals for not achieving desired outcomes in patients with leg lengthening as compared to patients without leg length inequality; (A) denotes lengthening of 1 cm, and (B) lengthening of >1 cm.

Figure 2 shows odds ratios and adjusted 95% confidence intervals for not achieving desired outcomes due to leg lengthening after THA. The odds ratio for not being able to walk for an hour was 1.70 (95% aCI 1.28-2.26, p_a_ < 0.001) for cases compared to controls (reference group), and the odds ratio for having hip pain at follow-up was 1.13 (95% aCI 0.78-1.64, p_a_ = 1). The odds ratio for limping was 2.08 (95% aCI 1.55-2.80, p_a_ < 0.001). Also, the odds ratio for not achieving excellent patient satisfaction was 1.67 (95% aCI 1.23-2.28, p_a_ < 0.001). Hence, the three outcomes walking capacity, limping and patient satisfaction were all significantly influenced by leg lengthening, whereas pain alleviation was not significantly affected.

#### Subgroup analysis leg lengthening

To estimate the influence of the extent of leg lengthening on patient outcomes, we compared two subgroups of cases with the controls. In subgroup A (lengthening = 1 cm), 53% (213 of 405 patients) were not able to walk over 60 minutes without support, while in subgroup B (lengthening > 1 cm) 62% (45 of 73 patients) could not do so. Hip pain was reported in 20% (82 patients) of subgroup A and in 19% (14 patients) of subgroup B. Limping was seen in 49% (198 patients) of subgroup A and in 68% (50 patients) of subgroup B. Patient satisfaction was not excellent in 35% (143 patients) of subgroup A and in 47% (34 patients) of subgroup B.

Odds ratios and adjusted 95% confidence intervals for not achieving the desired outcomes in the two subgroups are shown in Figure 2. The odds ratio for walking less than 60 minutes with a lengthened leg versus equally long legs was 1.61 (95% aCI 1.19-2.17, p_a_ < 0.001) for subgroup A and 2.33 (1.18-4.57, p_a_ = 0.002) for subgroup B. The odds ratio for having pain was 1.14 (95% aCI 0.77-1.70, p_a_ = 1) for subgroup A and 1.07 (0.47-2.45, p_a_ = 1) for subgroup B. The odds ratio for limping was 1.85 (95% aCI 1.35-2.54, p_a_ < 0.001) for subgroup A and 4.20 (2.08-8.50, p_a_ < 0.001) for subgroup B. The odds ratio for not achieving excellent patient satisfaction was 1.55 (95% aCI 1.12-2.16, p_a_ = 0.001) for subgroup A and 2.48 (95% aCI 1.28-4.80, p_a_ = 0.001) for subgroup B. Hence, there was a direct relationship between the extent of leg lengthening and the odds of not achieving the desired outcomes. Again, pain alleviation was not significantly affected by leg lengthening.

### Leg shortening

#### Patient characteristics leg shortening

A total of 275 cases with leg shortening were identified, comprising 245 patients with a shortening of 1 cm and 30 with a shortening of more than 1 cm. To these cases, 825 controls were matched. Similar to the first study, propensity-score matching achieved a nearly equal distribution of available pre-operative patient characteristics between cases and controls (Table 2).

#### Influence of leg shortening on patient outcomes

At follow-up, 48% (133 patients) in the case group were not able to walk for more than 60 minutes without support, compared to 43% (356 patients) in the control group. Hip pain was reported in 29% (79 patients) of cases and in 20% (166 patients) of controls. Limping was seen in 63% (172 patients) of cases and 39% (322 patients) of controls. Also, 48% (132 patients) in the case group did not rate their subjective patient satisfaction as excellent, as compared to 30% (248 patients) of the controls.

**Figure 3:**
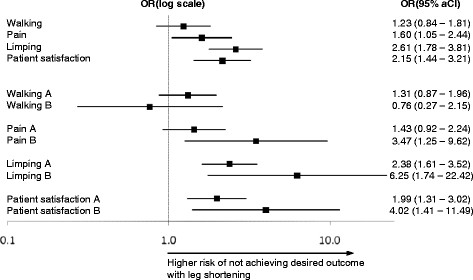
Odds ratios and 95% adjusted confidence intervals for not achieving desired outcomes in patients with leg shortening as compared to patients without leg length inequality; (A) denotes shortening of 1 cm, and (B) shortening of >1 cm.

Figure 3 shows odds ratios and adjusted 95% confidence intervals for not achieving desired outcomes due to leg shortening after THA. The odds ratio for not being able to walk for an hour was 1.23 (95% aCI 0.84-1.81, p_a_ = 0.527) for cases compared to controls, and the odds ratio for having hip pain at follow-up was 1.60 (95% aCI 1.05-2.44, p_a_ = 0.009). The odds ratio for limping was 2.61 (95% aCI 1.78-3.81, p_a_ < 0.001). Also, the odds ratio for not achieving excellent patient satisfaction was 2.15 (95% aCI 1.44-3.21, p_a_ < 0.001). Hence, the three outcomes hip pain, limping and patient satisfaction were all significantly influenced by leg shortening, whereas walking capacity was not significantly affected.

#### Subgroup analysis leg shortening

To estimate the influence of the extent of leg shortening on patient outcomes, we compared two subgroups of cases with the controls. In subgroup A (shortening = 1 cm), 50% (122 of 245 patients) were not able to walk over 60 minutes without support, while in subgroup B (shortening > 1 cm) 37% (11 of 30 patients) could not do so. Hip pain was reported in 27% (65 patients) of subgroup A and in 47% (14 patients) of subgroup B. Limping was seen in 60% (148 patients) of subgroup A and in 80% (24 patients) of subgroup B. Patient satisfaction was not excellent in 46% (113 patients) of subgroup A and in 63% (19 patients) of subgroup B.

Figure 3 depicts odds ratios and 95% confidence intervals for not achieving desired outcomes in the two subgroups. The odds ratio for walking less than 60 minutes with a leg shortening versus equally long legs was 1.31 (95% aCI 0.87-1.96, p_a_ = 0.278) for subgroup A and 0.76 (0.27-2.15, p_a_ = 1) for subgroup B. The odds ratio for having pain was 1.43 (95% aCI 0.92-2.24, p_a_ = 0.108) for subgroup A and 3.47 (1.25-9.62, p_a_ = 0.003) for subgroup B. The odds ratio for limping was 2.38 (95% aCI 1.61-3.52, p_a_ < 0.001) for subgroup A and 6.25 (1.74-22.42, p_a_ < 0.001) for subgroup B. The odds ratio for not achieving excellent patient satisfaction was 1.99 (95% aCI 1.31-3.02, p_a_ < 0.001) for subgroup A and 4.02 (1.41-11.49, p_a_ = 0.001) for subgroup B. Hence, with the exception of walking capacity there was a direct relationship between the extent of leg shortening and the odds of not achieving the desired outcomes, albeit not significant in pain alleviation.

## Discussion

### Summary of findings

The two cohort studies reported here separately investigated the influence of leg lengthening and leg shortening after total hip arthroplasty with respect to physical function, assessed by objective factors with arbitrarily defined desired outcomes, and patient satisfaction. We showed that 1 cm of postoperative leg lengthening was significantly associated with walking capacity, limping and patient satisfaction but not pain alleviation. There was a direct relationship between the extent of leg lengthening and the odds of not achieving the desired outcomes. One centimetre of leg shortening had a significant impact on limping, pain alleviation and patient satisfaction but not on walking capacity. We could also show that there is a relationship between the extent of leg shortening and the odds of limping and not achieving a good patient satisfaction. Leg shortening, although less common, appears to have a stronger impact on the desired outcomes and on patient satisfaction than leg lengthening.

### Leg lengthening

Leg length inequality of a minor degree is a relatively common complication of THA [12,13]. Reported incidences range widely from 5% [14,15] to almost 95%, depending on the definition of leg length inequality [13,16]. Early assessments of the complications of THA showed that a lengthening of the operated side is the most common form of leg length inequality. In 1978, Williamson showed that 144 of 150 patients with leg length inequality presented with a lengthening, while only 6 had a shorter leg after the intervention [16]. The average lengthening in this group was 15.9 mm. One factor contributing to the more frequent reports of leg lengthening than shortening could be the different subjective perception of patients. Konyves et al. found that patients were more likely to notice a lengthened leg compared to those with leg shortening [13]. The consequences of leg length inequality have been widely debated in the orthopaedic literature, with only one article stating that it has no impact on the outcome of THA [17]. Williamson found that five patients developed incomplete sciatic nerve palsy, although no statistically significant association to leg lengthening was apparent. Other authors assumed leg lengthening to be a major cause of postoperative neuronal dysfunction in operated legs. Friberg found that 228 of 1157 patients with back or hip pain also showed sciatic symptoms, with almost 80% radiating into the longer leg [18]. Della Valle found an incidence of peripheral nerve injury of 0-3% and a strong association between a lengthening of more than four centimetres and an increased risk of neuronal injury during THA [19]. Another possible adverse effect of leg lengthening after THA may be aseptic loosening of the prosthesis, however the causality between leg lengthening and aseptic loosening is difficult to establish. Visuri prospectively studied 405 patients and found the rate of aseptic loosening to be 23.9% in cases with a lengthening of 1–2 cm and increasing to 50% in cases with a lengthening of 3-5 cm [20]. Gurney et al. studied effects of leg lengthening on gait economics and muscular activity in the lower extremities [21]. They equipped subjects with different levels (2, 3 or 4 cm) of shoe lifts to simulate leg lengthening and asked them to walk on treadmills. Leg lengthening had a significant effect on oxygen consumption, activity of the quadriceps femoris muscle and the subjectively perceived effort.

Only few articles consider the patient perspective of leg length inequality, and we found no more than two publications concerned with a rating of patient satisfaction [22,23]. However, none of these studies combined a discussion of objective functional outcomes and subjective satisfaction in a large patient sample.

### Leg shortening

We found only a few studies on the impact of leg shortening on health outcomes. Edeen stated that patients with leg shortening generally tended to limp more than patients with leg lengthening [23]. Austin reported that leg shortening leads to lax soft tissues which increases the risk of hip dislocation, in contrast to leg lengthening which tends to tighten the soft tissues [24]. Similarly, Suh et al. explained that shortening of an operated leg can impair abduction and increase the likelihood of dislocation [25]. In accordance to this, Abraham found that shortening of the leg leads to impaired abductor function and hence to overall poor outcome, and in particular to an increased risk of dislocation [26]. Gore et al. showed that shortening of the limb leads to length-tension disadvantage for the abductor muscles and subsequent decrease in strength [27]. Tallroth et al. found that osteoarthritis was more common in the hip of a longer leg, which raises the question if shortening of the operated leg might predispose to osteoarthritis in the hip of the contralateral leg [28]. Therefore, even though leg shortening is less common than leg lengthening after THA, it seems to have a greater impact on patient outcome. This finding is in accordance with the results of our two studies which revealed a higher chance for patient dissatisfaction caused by leg shortening than by lengthening.

### Weaknesses and strengths

The validity of assessed outcomes in this study depended on the accuracy of the physicians completing the follow-up forms; they were often the same surgeons who had previously carried out the intervention and therefore could be subject to examiner bias. Different clinical measurement methods of leg length inequality exist, ranging from clinical assessment, e.g. using blocks of known thickness on which patients stand with the shorter leg to equalise pelvic imbalance, to more sophisticated radiological methods, e.g. bilateral comparison of the distance between the centre of rotation and the tip of the greater trochanter [5,14,29-31]. Unfortunately, no information was recorded on IDES forms regarding the measurement method used by participating surgeons. This lack of a standardised method to measure leg length inequality may have led to observer bias. However, leg length inequality is a non-desired outcome and the fact that surgeons recorded it in a voluntary registry allows the conclusion that in these cases a clearly visible and relevant limb length difference was present, not just a radiographic measurement result. In addition, the crudeness of the centimetre scaling helped to keep the measurements simple, yet sufficiently precise.

Clark stated that clinical measurements do not appropriately distinguish between functional leg length inequality caused by contractures and real inequality [5]. In addition, Hoikka found that true leg length measured on antero-posterior roentgenograms was misleading and correlated poorly with intraoperative alterations of leg length and post-operative pelvic tilt because it does not pay respect to the position of the hip joint on the pelvic wall [30]. Furthermore, according to Clark and Ranawat, contractures should normally disappear by 6 months post-op, hence in our study they should not have influenced the clinical measurements at the follow-up examinations performed 2 years after the intervention [3,5,32]. We made no exclusions based upon status of contralateral hip, locomotor system or comorbid conditions (i.e. atherosclerosis, heart condition, etc.), which could all potentially influence outcomes like walking endurance. As osteoarthritis of the hip is commonly diagnosed in elderly patients who almost inevitably suffer from comorbidities, we consciously included all patients to increase the generalisability of results.

### Implications for clinical practice and future research

Careful preoperative planning is crucial for avoiding leg length inequality after THA and the related consequences. The great number of articles about this topic reflects the significance and magnitude of the problem [3,25,26,29-31,33-37]. The consequences of leg length inequality on other parts of the musculoskeletal system, particularly the contralateral hip and lower back, still need clarification. The investigation of all these outcomes requires prospective study designs. Randomised comparisons are clearly not feasible here, not only because it would be unethical but also due to the large sample sizes that would be required to obtain robust estimates in the relatively rare events of leg length inequality.

## Conclusion

This study showed that leg lengthening after THA is significantly associated with walking capacity, limping and patient satisfaction, while leg shortening is associated with hip pain, limping and patient satisfaction. Leg lengthening seems to be more common than shortening; however, shortening has a stronger impact on the desired outcomes and on patient satisfaction.

## Competing interests

The authors declare that they have no competing interests.

## Authors’ contributions

CR conceived the research idea, contributed to the selection of research methods, interpreted the findings and drafted the manuscript. RV and LB contributed to the interpretation of findings and drafting of the manuscript, as part of their medical thesis. DD supervised the statistical analyses and participated in the interpretation of the findings and the drafting of the manuscript. LPS developed the research proposal, selected the research methods, conducted the statistical analyses, and participated in the interpretation of findings and the drafting of the manuscript. All authors read and approved the final manuscript.

## Source of funding

There was no external source of funding for the current study.

## Pre-publication history

The pre-publication history for this paper can be accessed here:

http://www.biomedcentral.com/1471-2474/13/95/prepub
